# An ACE2-Alamandine Axis Modulates the Cardiac Performance of the Goldfish *Carassius auratus* via the NOS/NO System

**DOI:** 10.3390/antiox11040764

**Published:** 2022-04-12

**Authors:** Mariacristina Filice, Rosa Mazza, Sandra Imbrogno, Olga Mileti, Noemi Baldino, Amilcare Barca, Gianmarco Del Vecchio, Tiziano Verri, Alfonsina Gattuso, Maria Carmela Cerra

**Affiliations:** 1 Department of Biology, Ecology and Earth Sciences, University of Calabria, Arcavacata, 87036 Rende, Italy; rosa.mazza@unical.it (R.M.); sandra.imbrogno@unical.it (S.I.); maria_carmela.cerra@unical.it (M.C.C.); 2 Department of Information, Modeling, Electronics and System Engineering, University of Calabria, Arcavacata, 87036 Rende, Italy; o.mileti@dimes.unical.it (O.M.); noemi.baldino@unical.it (N.B.); 3 Department of Biological and Environmental Sciences and Technologies, University of Salento, 73100 Lecce, Italy; amilcare.barca@unisalento.it (A.B.); gianmarco.delvecchio@unisalento.it (G.D.V.); tiziano.verri@unisalento.it (T.V.)

**Keywords:** ACE2, almandine, NOS/NO system, heart, teleost, *Carassius auratus*

## Abstract

Alamandine is a peptide of the Renin Angiotensin System (RAS), either generated from Angiotensin A via the Angiotensin Converting Enzyme 2 (ACE2), or directly from Ang-(1–7). In mammals, it elicits cardioprotection via Mas-related G-protein-coupled receptor D (MrgD), and the NOS/NO system. In teleost fish, RAS is known to modulate heart performance. However, no information is available on the presence of a cardioactive ACE2/Alamandine axis. To fill this gap, we used the cyprinid teleost *Carassius auratus* (goldfish) for in silico and in vitro analyses. Via the NCBI Blast P suite we found that in cyprinids ace2 is phylogenetically detectable in a subcluster of proteins including ace2-like isoforms, and is correlated with a hypoxia-dependent pathway. By real-time PCR, Western Blotting, and HPLC, ACE2 and Alamandine were identified in goldfish heart and plasma, respectively. Both increased after chronic exposure to low O_2_ (2.6 mg O_2_ L^−1^). By using an ex-vivo working goldfish-heart preparation, we observed that in vitro administration of exogenous Alamandine dose-dependently stimulates myocardial contractility starting from 10^−11^ M. The effect that involved Mas-related receptors and PKA occurred via the NOS/NO system. This was shown by exposing the perfused heart to the NOS inhibitor L-NMMA (10^−5^ M) that abolished the cardiac effect of Alamandine and was supported by the increased expression of the phosphorylated NOS enzyme in the extract from goldfish heart exposed to 10^−10^ M Alamandine. Our data are the first to show that an ACE2/Alamandine axis is present in the goldfish *C. auratus* and, to elicit cardiac modulation, requires the obligatory involvement of the NOS/NO system.

## 1. Introduction

The octapeptide Angiotensin II (AngII) has long been considered the major end-effector of the Renin Angiotensin System (RAS), the enzymatic axis activated by the renal release of renin, and that involves the angiotensin-converting enzyme (ACE), plus other parallel enzymes. In all vertebrate classes, AngII, via specific receptors (AT1R and/or AT2R), acts as a master regulator of the idromineral and cardiovascular homeostasis, showing high evolutionary conservation and a long phylogenetic history [1,2,3].

In the past few years, the classical view of the RAS as a linear cascade has been changed by the characterization of novel components, including new peptides, enzymes, and receptors. The existence of an alternative axis, which includes the angiotensin-converting enzyme 2 (ACE2; [4,5]), Ang-(1–7) [6], and the Mas receptor (MasR) [7], is now largely demonstrated. This alternative axis in mammals is recognized as counteracting many effects of the classic ACE/AngII/AT1R (reviewed in [8]). Very recently, a new peptide with a structure similar to Ang-(1-7) has been detected in the plasma of rats and humans, and in the aorta of mice and rats [9]. This peptide, named Alamandine, can be formed directly from Ang-(1–7), via decarboxylation of Asp1 into Ala1, or from angiotensin A (Ang A) via the ACE2-mediated catalysis of the C-terminal Phe residue. By binding to Mas-related G-protein-coupled receptor D (MrgD), Alamandine exerts antifibrotic effects on isoproterenol-treated rats, antihypertensive effects on spontaneously hypertensive rats, and central cardiovascular effects if injected into the caudal ventrolateral medulla or rostral ventrolateral medulla of rats (depressor and pressor effects, respectively) [9]. Moreover, the activation of the Alamandine/MrgD axis elicits cardioprotection against ischemia/reperfusion (I/R) injury in rodents [10]. Specifically, in rat ventricles exposed to I/R insult, pre-exposure to Alamandine improved the reperfusion-induced ventricular hemodynamics, decreased infarct size, and reduced lactate dehydrogenase release. This was associated with an improved activation of antioxidant enzymes and a decreased expression of pro-apoptotic markers [10]. All the above evidence proposes the Alamandine/MrgD receptor as an additional beneficial axis which, similarly to that formed by Ang-(1–7)/ACE2 and Mas receptors, counterbalances the actions mediated by AngII/ACE/AT1. This is supported by data from Jesus et al., showing that Alamandine, acting via the MrgD receptor, activates the AMPK/NO pathway to protect adult mice cardiomyocytes from ANGII-mediated hypertrophy [11].

In non-mammalian vertebrates, information is available on the presence and function of an analogue of the mammalian RAS characterized by cardiovascular activity [1,12]. AngII is known to exert both direct and indirect (i.e., via cardiac adrenoceptors) stimulatory effects on the heart of the American eel *Anguilla rostrata* and of the trout *Oncorhynchus mykiss* [13,14], and a cardioinhibitory activity in the European eel *Anguilla anguilla* [15]. Moreover, in eel [16,17] and zebrafish [18], long-term exposure to AngII elicits cardiac remodeling by increasing muscle mass and eliciting fibrosis. Interestingly, the identification of ACE activity in the ventricle of various teleost species [19], of immunoreactive AngII-like material in the heart of the Antarctic teleost *Champsocephalus gunnari* [20], and of AngII binding sites in the cardiac extracts of trout [21] and eel [16], support the presence of a tissue RAS in the teleost heart.

As in mammals, also in teleost the RAS pathway was recently enriched by the identification of different Ang peptides. Two isoforms of AngII, [Asn1]- and [Asp1]-AngII, were found in trout (brain and blood) [22,23], and eel (blood) [24], and the AngII-derived peptides, AngIII [AngII (2–8)] and AngIV [AngII (3–8)], were detected in trout plasma [23]. However, to the best of our knowledge, the presence of Alamandine and its role in cardiovascular homeostasis received no attention in the teleost species.

By taking advantage of in vitro and in silico analyses we explored, in the goldfish *Carassius auratus*, the expression of a functional ACE2/Alamandine axis and its possible correlation with hypoxia. Investigations were also carried out to evaluate whether Alamandine can influence the basal cardiac hemodynamics, and if this requires the activation of the intracardiac NOS/NO system. Results from this study are the first to provide information on this limb of the RAS in a teleost fish, confirming the old evolutionary history and the importance of this enzymatic-hormonal system.

## 2. Materials and Methods

### 2.1. Animals

Goldfish (*C. auratus*; length = 12–16 cm; weight = 31.57 ± 1.87 g; means ± s.e.m.) specimens of both sexes were provided by local hatcheries. Fish were maintained at 18–21 °C in filtered and aerated water, 12 h light/dark cycle, and fed daily with commercial food. Animal care and experimental procedures were in accordance with the European Directive (2010/63/EU), and the Italian law (DL 27 January 1992, n.116), which did not require specific authorization for the used species by an ethics committee.

### 2.2. Hypoxia Exposure

Goldfish were randomly transferred to two 20 L experimental tanks (6 fish each time), and left to acclimate for at least 24 h. Subsequently, aquaria were covered with a Plexiglas lid and the water was continuously bubbled with nitrogen gas (hypoxia) or regular air (normoxia). Oxygen values were maintained at 2.6 ± 0.3 mg L^−1^ in the hypoxic experimental tank, and at 8.2 ± 0.4 mg L^−1^ in the normoxic one. Oxygen saturation in the aquaria was continuously monitored by an oxygen analyzer (Milwaukee, SM600, Szeged, Hungary). Hypoxia exposure was protracted for 4 days. At the end of the exposure period, animals were sacrificed after anesthesia with tricainemethanesulfonate (MS222; 0.2 g L^−1^) (Sigma Aldrich, Milan, Italy). A blood sample was taken from the caudal vessels with a heparinized syringe, transferred into Eppendorf tubes and centrifuged for 5 min at 8000 rpm. Plasma was used for Alamandine determination by HPLC. Hearts were isolated and stored at −80 °C for molecular analysis.

### 2.3. In Silico Analyses

#### 2.3.1. Protein Sequence Alignment

The Basic Local Alignment Search Tool (BLAST^®^, version BLAST+ 2.12.0, National Center for Biotechnology Information (US), Bethesda (MD)) software available from the NCBI platform was used to search for primary protein structure homology and alignment, with the BLASTP option (https://blast.ncbi.nlm.nih.gov/blast/Blast.cgi?PROGRAM=blastp&PAGE_TYPE=BlastSearch&LINK_LOC=blasthome*,* accessed on 15 October 2021). The ace2 reference protein sequence of *C. auratus* (Acc. N. XP_026131313.1) was matched with the reference proteins (“refseq_protein” database) from the subset of Teleostei (taxid: 32443).

#### 2.3.2. Protein-Protein Interaction Network

Analysis of the protein–protein interaction network (PPI) was performed with the use of the STRING online suite (version 11.0b, STRING CONSORTIUM 2022©, https://version-11-0b.string-db.org/, accessed on 15 April 2021) for PPI networks and functional enrichment analysis. A PPI network was constructed based on 14 proteins (number of nodes: 14; number of edges: 38; average node degree: 5.43; avg. local clustering coefficient: 0.623; expected number of edges: 2; PPI enrichment *p*-value: <1.0 × 10^−16^).

### 2.4. Alamandine Detection in Plasma

Alamandine content in goldfish plasma samples was determined using HPLC. Analyses were carried out on a Smartline HPLC system (Knauer, Berlin, Germany), equipped with a degasser, a pump, and a UV detector 2600. Chromatographic separation was performed by 250 mm × 4.6 mm i.d., with precolumn, C18 Ascentis (Supelco, Darmstadt, Germany), at 32 °C. The mobile phase composition was composed by 0.1%TFA in Water (Solvent A) and 0.1%TFA in 60% acetonitrile/water (Solvent B), at a flow rate of 1 mL/min. The gradient used in the investigation was 5–100% Solvent B in 40 min. The method used was according to Phoenix Pharmaceuticals, Inc. Absorbance spectra were recorded every 1 s, between 200 and 420 nm, with a bandwidth of 8 nm. Chromatograms were acquired at 254, 220 and 280 nm and the analysis was performed at 220 nm, this is the absorption maxima. The measurements were performed in duplicate. A calibration curve was set by analyzing several concentrations of Alamandine standard (Phoenix Pharmaceuticals, Inc. Burlingame, CA, USA) (see Figure S1 in the Supplementary Materials).

### 2.5. Isolated and In Vitro Perfused Working Heart

Goldfish, maintained in filtered and aerated water, were anesthetized with MS222; the heart was removed without the parietal pericardium, cannulated, and connected to a perfusion apparatus as previously described [25]. The perfused heart received Ringer’s solution (in mmol L^−1^: NaCl 124.9, KCl 2.49, MgSO_4_ 0.94, NaH_2_PO_4_ 1.0, Glucose 5.0, NaHCO_3_ 15.0, and CaCl_2_ 1.2, pH 7.7) from an input reservoir and pumped against an afterload pressure given by the height of an output reservoir. Saline was equilibrated with a mixture of 99.5% O_2_ and 0.5% CO_2_. Experiments were carried out at room temperature (18–20 °C). Pressures were measured with two MP-20D pressure transducers (Micron Instruments, Simi Valley, CA, USA) connected to a PowerLab data acquisition system and analyzed by using LabChart software, version 8 (ADInstruments Basile, Comerio, Italy). Pressures were corrected for cannula resistance. Cardiac output (CO) was collected over 1 min and weighed. Values were corrected for fluid density and expressed as volume measurements. Heart rate (HR, bpm) was obtained from the periodicity of pressure traces. Stroke volume (SV = CO/HR, mL/min/Kg) was used as a measure of ventricular performance. Ventricular stroke work (SW; mJ/g; (afterload-preload) × SV/ventricle mass) served as an index of systolic functionality.

### 2.6. Experimental Protocols

#### 2.6.1. Basal Conditions

The isolated and perfused goldfish heart was allowed to maintain a spontaneous rhythm for up to 15–20 min. For control conditions, afterload was set to 1.5 kPa, and CO to 10–14 mL min^−1^ kg^−1^ body mass, by appropriately adjusting output and filling pressure, respectively. Cardiac variables were simultaneously measured throughout the experiment. Hearts that did not stabilize within 20 min of perfusion were discarded.

#### 2.6.2. Drug Application

After stabilization, cardiac preparations were perfused with Ringer’s solution enriched with Alamandine at increasing concentrations (from 10^−12^ M to 10^−7^ M) to generate cumulative concentration–response curves. Cardiac variables were measured after 10 min of perfusion with each concentration of the drug.

To investigate the receptors involved in the Alamandine pathway, hearts were stabilized and then perfused with either the Mas antagonist D-Ala7-Ang-(1-7) A-779 (10^−11^ M), or the MrgD inhibitor D-Pro7-Ang-(1–7) (10^−12^ M) for 15–20 min followed by the perfusion with Alamandine (10^−10^ M) plus the correspective antagonist for an additional 20 min. The involvement of NO was evaluated in the presence of the NOS inhibitor L-NMMA (10^−5^ M). The role of PKA as the putative intracellular activator of eNOS was tested by using the PKA inhibitor KT5720 (10^−7^ M).

Inhibitor concentration was selected on the basis of preliminary dose–response curves, as the highest dose that did not significantly affect the goldfish basal cardiac performance.

#### 2.6.3. Drugs and Chemicals

Alamandine was purchased from Phoenix Pharmaceuticals, Inc.; D-Pro7-Ang(1-7) and A-779 were obtained from Bachem; L-NMMA was obtained from Sigma–Aldrich; and KT5720 was obtained from Santa Cruz Biotechnology. All drugs were prepared in double-distilled water, with the exception of KT5720 prepared in DMSO. All dilutions were made in the Ringer’s solution immediately before use.

### 2.7. RNA Extraction

Isolated tissues (heart, muscle, liver, intestine, brain, gills) from goldfish acclimated to normoxia, and heart from goldfish acclimated to 4-days hypoxia, were stored in RNALater^TM^ (Ambion-ThermoFisher Scientific, Milan, Italy), and processed for RNA extraction by using the AllPrep DNA/RNA/Protein mini kit (Qiagen, Milan, Italy) protocol and reagents, according to the manufacturer’s instructions. Total tissue lysis was performed with the AllPrep lysis buffer and by using a mini homogenizer (Thermo-Fisher Scientific, Milan, Italy). RNA aliquots were stored in RNase-free conditions at −80 °C until use. RNA concentrations were calculated by spectrophotometry, and λ260/λ280 ratios were checked to evaluate protein contamination. All RNA-extracted samples were loaded onto agarose gel for qualitative analysis.

### 2.8. Primer Design and Real-Time PCR

For each investigated gene, goldfish mRNA reference sequence was collected from the GenBank database (https://www.ncbi.nlm.nih.gov/, accessed on 28 January 2022). The exon–intron structure was analyzed using the Splign mRNA-to-genomic alignment tool (https://www.ncbi.nlm.nih.gov/sutils/splign/splign.cgi, accessed on 28 January 2022) and forward and reverse primers were designed by selecting oligonucleotide sequences in adjacent exons, so as to avoid possible genomic amplicons. The program AmplifX version 1.5.4 by Nicolas Jullien; Aix-Marseille Univ, CNRS, INP, Inst Neurophysiopathol, Marseille, France-https://inp.univ-amu.fr/en/amplifx-manage-test-and-design-your-primers-for-pcr (accessed on 4 April 2022) was used to test PCR size, GC content, end stability, and self-/cross-dimer formation of the selected oligonucleotides which were purchased from Metabion (Metabion International, Germany). The sequences of primers used for qPCR assays are reported in Table 1. For each extracted RNA, reverse transcriptions were performed on 0.25–1 μg total RNA using the Bio-Rad iScript Select cDNA Synthesis kit (Bio-Rad, Italy), according to the manufacturer’s instructions, using random primers. Before qPCR analysis, each gene-specific primer pair was tested for efficiency, according to the amplification efficiency parameters for genes of interest and internal controls proposed by Schmittgen and Livak [26] and as previously described in [18]. The qPCR was performed using the iQ SYBR Green Supermix protocol (Bio-Rad) with a CFX96 Touch Real Time PCR detection system (Bio-Rad). For quantitative gene expression analysis, 28S was used as internal control. Gene expression relative quantification was assessed by analyzing the output threshold values (Ct) according to the comparative Ct method [26]; qPCR data were shown as 2-ΔCT mean values, which are taken as proportional to the amount of the detected target mRNA. ΔCt values (ΔCt = target gene Ct—housekeeping gene Ct) were obtained from three different rounds of qPCR for both the target mRNA and the 28S, on cDNAs from independent biological replicates per treatment condition.

### 2.9. Western Blot and Densitometric Analysis

Hearts were homogenized in an ice-cold homogenization buffer (250 mmol L^−1^ sucrose, 30 mmol L^−1^ Tris, 1 mmol L^−1^ EDTA, 1% SDS, pH 7.4), containing a mixture of protease inhibitors (1 mmol L^−1^ aprotinin, 20 mmol L^−1^ phenylmethylsulfonyl fluoride and 200 mmol L^−1^ sodium ortho-vanadate). Homogenates were centrifuged at 10,000 g for 10 min at 4 °C to remove tissue debris. Protein concentration in the supernatant was determined using Bradford reagent (Sigma–Aldrich, Milan, Italy), according to the manufacturer’s instructions. Western Blotting was performed as previously described [27]. Briefly, a 60 μg protein sample for each homogenate was separated on SDS/10% and SDS/8% polyacrylamide gels and electroblotted onto a nitrocellulose membrane (GE Healthcare, Milan, Italy). Blots were blocked in TBS-T containing 5% non-fat dry milk and incubated overnight at 4 °C with either mouse monoclonal antibody against ACE2 (cat# Sc-73668; dilution 1:500), or rabbit polyclonal antibodies directed against Akt1/2/3 (Santa Cruz Biotechnology, cat# Sc-8312), pAkt1/2/3-Ser473 (cat# Sc-7985-R), AMPKα (Cell Signaling Technology, cat# 5831; dil 1:500), pAMPKα (Thr172) (Cell Signaling Technology, cat# 2535; dil 1:500), eNOS (cat# N3893), or goat polyclonal antibody directed against pNOS3-Ser1177 (cat# Sc-12972; dil. 1:500). Glyceraldehyde-3-Phosphate Dehydrogenase (GAPDH) (cat# Sc-47724; dil 1:20000) or β-actin (cat# Sc-69879; dil. 1:2000) antibodies were used as loading control. Peroxidase-linked secondary antibodies were diluted to 1:1000 in TBS-T containing 5% non-fat dry milk, and incubated for 1 h at RT. All antibodies were from Santa Cruz Biotechnology (Santa Cruz, CA, USA) with exception for eNOS which was from Sigma Aldrich (Milan, Italy). Immunodetection was performed using an enhanced chemiluminescence kit (ECL PLUS, GE Healthcare, Milan, Italy). Autoradiographs were scanned to obtain arbitrary densitometric units. Experiments were performed in triplicate; results were expressed as means ± s.e.m. of absolute values.

### 2.10. Statistics and Calculations

Alamandine quantification in blood samples was expressed as pmol/mL.

Hemodynamic data were expressed as means ± s.e.m. of percentage changes obtained from individual experiments. Statistical analysis was performed by using two-way or one-way ANOVA, followed by either Bonferroni’s or Dunnett’s post-test. Differences were considered statistically significant at *p* < 0.05.

Densitometric analyses were expressed as means ± s.e.m. of absolute values from individual experiments; statistics were assessed by two-tailed unpaired *t*-test. Significance was concluded at *p* < 0.05.

For real-time PCR data, values were expressed as means ± s.e.m.; statistical analysis was performed after the 2-ΔCt transformation [26]. Statistics were assessed by one-way ANOVA followed by a Sidak’s multiple comparison test.

GraphPad Prism software, version 4.02 (GraphPad Software Inc., San Diego, CA, USA), was used for all statistical analyses.

## 3. Results

### 3.1. Identification of ACE2 and Alamandine

By using the NCBI Blast P suite (see methods Section 2.3.1 for details), we observed that the goldfish ace2 protein product (NCBI Acc. No. XP_026131313.1) is phylogenetically located in a subcluster of proteins from other teleost species (namely, the cyprinid *Puntigrus tetrazona*, *Cyprinus carpio*, *Sinocyclocheilus rhinocerous*, *Sinocyclocheilus grahami*, and *Sinocyclocheilus anshuiensis*), which includes both ace2 and ace2-like isoforms (see Supplementary Figure S2).

**Figure 1:**
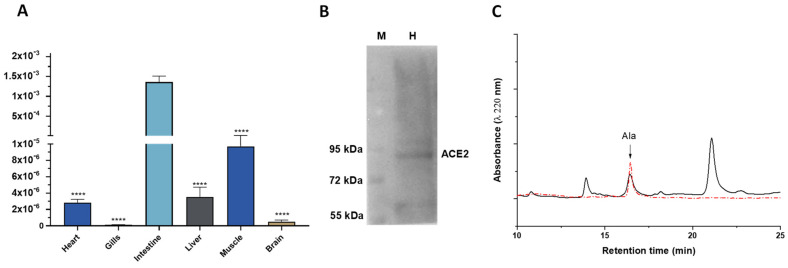
(**A**) ace2 mRNA expression levels in goldfish *C. auratus* tissues. The amounts of target mRNA are calculated as 2-ΔCt mean values obtained from the output Ct values of two rounds of real-time PCR assays for each of three independent biological replicates. Statistics were assessed by one-way ANOVA followed by a Sidak’s multiple comparison test (**** *p* < 0.0001); (**B**) Representative immunoblotting of ACE2 expression in the goldfish heart. M: marker; H: heart; (**C**) Representative HPLC chromatogram showing Alamandine (Ala) elution from *C. auratus* plasma compared to Alamandine standard (red dotted line).

Quantitative real-time PCR showed that the ACE2 gene product is present in several goldfish tissues. Expression of ace2 mRNA was detected in goldfish heart, gills, intestine, liver, muscle, and brain. As shown in Figure 1A, higher levels were detected in intestine; compared to intestine, significantly lower levels were found in heart, liver, and muscle, and faint although detectable levels were found in gills and brain.

A basal cardiac ACE2 protein expression was also confirmed by Western Blotting analysis, which revealed an immunoreactive band corresponding to the approximate molecular mass of ACE2 (92 kDa) (Figure 1B).

HPLC analysis performed on goldfish plasma samples showed the presence of a peptide with elution properties comparable with the Alamandine standard (Figure 1C). Quantification of the peak area revealed a basal concentration of the peptide ranging between 19.3 and 34.6 pmol/mL (27 ± 2.8; mean ± s.e.m.).

### 3.2. Effects of Alamandine on the Basal Cardiac Performance

**Figure 2:**
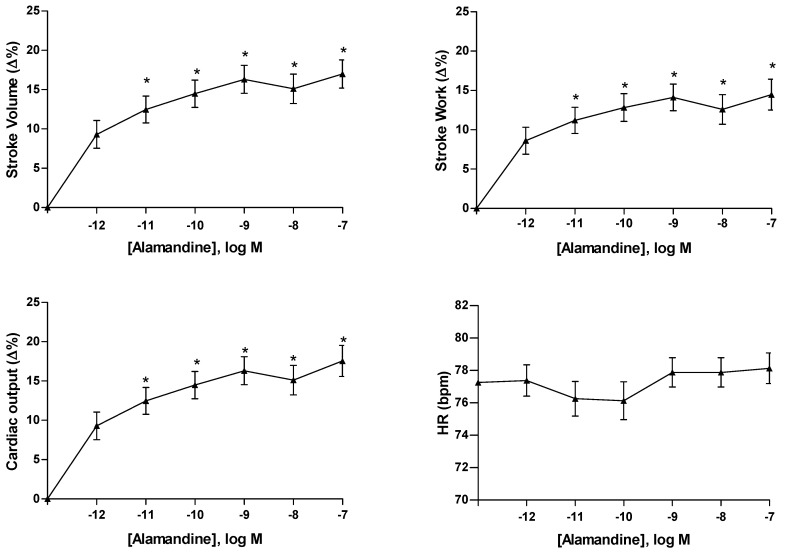
Cumulative concentration–response curves of Alamandine on SV, SW, CO, and HR in isolated and perfused working goldfish *C. auratus* heart. Percentage changes were evaluated as means ± s.e.m. of six experiments. Significance of difference from control values was assessed by repeated measures ANOVA, followed by Dunnett’s post-test; * *p* < 0.05.

The effects of Alamandine on cardiac hemodynamics were analyzed by exposing the in vivo, spontaneously beating, isolated and perfused working heart of the adult goldfish to exogenous Alamandine. Concentration–response curves obtained in the presence of Alamandine from 10^−12^ to 10^−7^ M showed that the peptide dose-dependently increases SV, CO and SW, thus acting as a positive inotrope (Figure 2). The percentage of the increment, compared to the basal values obtained before peptide administration (see Table 2), was significant, starting from 10^−11^ M (SV: 12.46 ± 4.28%; SW: 11.20 ± 4.08%; CO: 12.46 ± 4.18%) to 10^−7^ M (SV: 16.99 ± 4.39%; SW: 14.63 ± 4.8%; CO: 17.55 ± 4.84%) and occurred without significant changes of the HR (10^−11^ M: 76.25 ± 2.6 bpm; 10^−7^ M: 78.13 ± 2.32 bpm) (Figure 2).

### 3.3. Receptors

**Figure 3:**
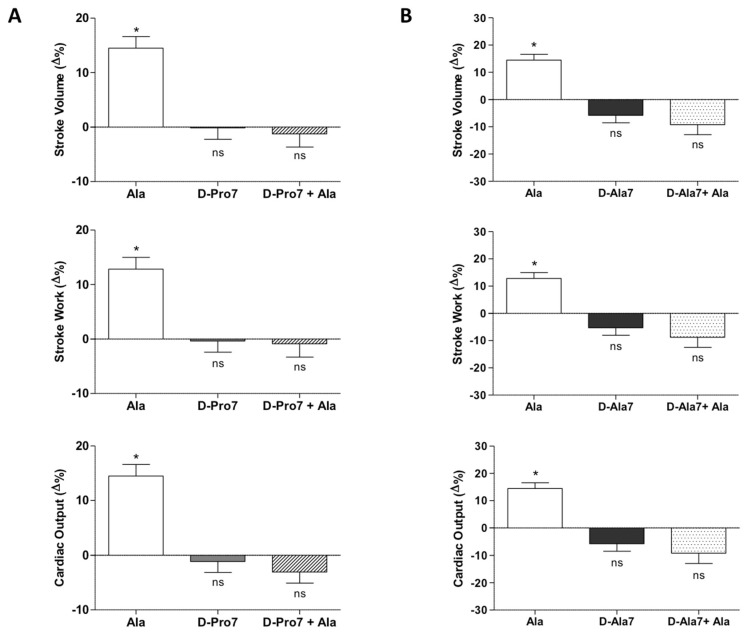
Effects of Alamandine (10^−10^ M) before and after treatment with D-Pro7 (10^−12^ M) (**A**) or D-Ala7 (10^−12^ M) (**B**) on SV, SW, and CO of the isolated and perfused goldfish heart. Percentage changes were evaluated as the mean ± s.e.m. of five experiments for each group. Statistical analysis was assessed by either repeated measures ANOVA followed by Dunnett’s post-test (* *p* < 0.05: Ala vs. control), or two-way ANOVA followed by Bonferroni post-test (ns = not-significant: D-Pro7 or D-Pro7 + Ala vs. control; D-Ala7 or D-Ala7 + Ala vs. control; D-Pro7 vs. D-Pro7 + Ala; D-Ala7 vs. D-Ala7 + Ala).

To analyze the receptor involved in the Alamandine-induced cardiostimulation in the goldfish, in vitro heart preparations were perfused with Alamandine (10^−10^ M) in the presence of D-Pro7-Ang-(1-7) (10^−12^ M), antagonist of the MrgD and Mas receptors. We found that the positive effect of Alamandine on SV, SW, and CO was abolished by the antagonist (Figure 3A). To further discriminate the receptor type involved in Alamandine cardio-activity, perfused hearts were exposed to the selective Mas receptor antagonist D-Ala7-Ang-(1-7) (A779; 10^−11^ M). In this case, the effect of Alamandine on cardiac parameters was also suppressed (Figure 3B).

### 3.4. Role of the NOS/NO System in the Alamandine-Induced Enhanced Contractility

**Figure 4:**
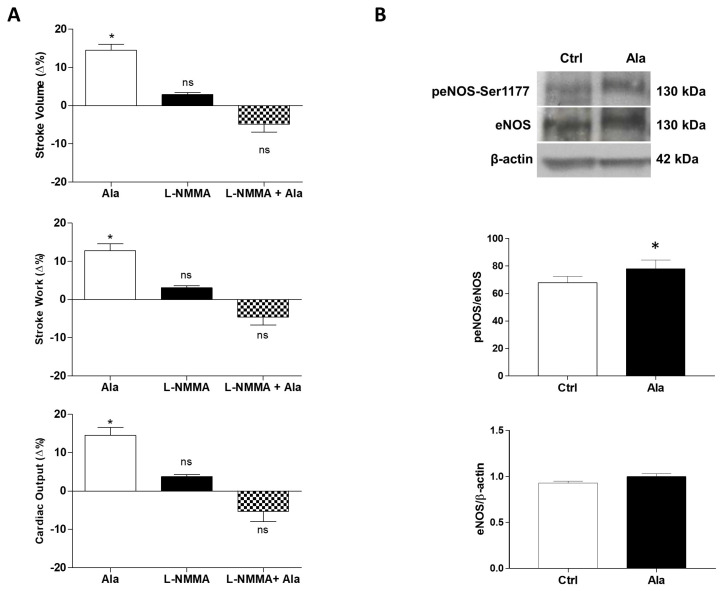
(**A**) Effects of Alamandine (10^−10^ M) before and after treatment with L-NMMA (10^−5^ M) on SV, SW, and CO of the isolated and perfused goldfish heart. Percentage changes were evaluated as the mean ± s.e.m. of seven experiments. Statistical analysis was assessed by repeated measures ANOVA followed by Dunnett’s post-test (* *p* < 0.05: Ala vs. control), or two-way ANOVA followed by Bonferroni post-test (ns = not-significant: L-NMMA or L-NMMA + Ala vs. control; L-NMMA vs. L-NMMA + Ala); (**B**) Representative immunoblot and densitometric analysis of eNOS/β-actin and peNOS/eNOS ratio in control hearts and in hearts treated with Alamandine (10^−10^ M). Data were expressed as means ± s.e.m. of absolute values from individual experiments (*n* = 4). Statistics were assessed by two-tailed unpaired *t*-test (* *p* < 0.05).

The role of NO as a major coordinator of many protective cascades is well established in mammals and in non-mammalian vertebrates [28]. It has been reported in rodent cardiac cells that Alamandine, by binding to MrgD, enhances cardiomyocyte contractility through a NO-dependent pathway [29]. To investigate whether in the goldfish the Alamandine-induced cardiac-stimulation is related to the production of NO, ex vivo heart preparations were pretreated with the NOS inhibitor L-NMMA (10^−5^ M). The NOS inhibitor abolished the Alamandine-dependent increase of SV, SW, and CO (Figure 4A), supporting an Alamandine-dependent NOS activation. This is reinforced by Western Blotting analyses performed by using mammalian anti-eNOS and anti-peNOS antibodies, on homogenates of goldfish hearts exposed to Alamandine 10^−10^ M, that showed an increased expression of a phosphorylated eNOS-like isoform in hearts exposed to the peptide, with respect to control hearts (Figure 4B).

**Figure 5:**
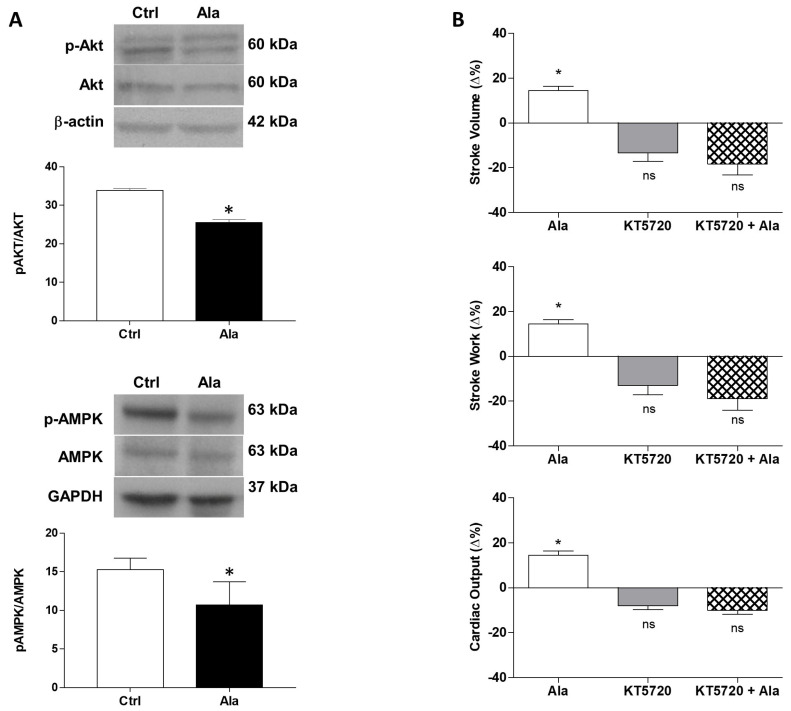
(**A**) Representative immunoblot and densitometric analysis of pAkt/Akt and pAMPK/AMPK in control hearts and in hearts treated with Alamandine (10^−10^ M). Data were expressed as means ± s.e.m. of absolute values from individual experiments (*n* = 3). Statistics were assessed by two-tailed unpaired *t*-test (* *p*< 0.05); (**B**) Effect of Alamandine (10^−10^ M) before and after treatment with KT5720 (10^−7^ M) on SV, SW, and CO of the isolated and perfused goldfish heart. Percentage changes were evaluated as the mean ± s.e.m. of five experiments. Statistical analysis was assessed by either repeated measures ANOVA followed by Dunnett’s post-test (* *p* < 0.05: Ala vs. control), or two-way ANOVA followed by Bonferroni post-test (ns = not-significant: KT5720 or KT5720 + Ala vs. control; KT5720 vs. KT5720 + Ala).

To further investigate the signaling pathways involved in the Alamandine-induced NOS activation, we assessed the phosphorylation of both protein kinase B (Akt), a key protein involved in NO production [30], and of AMP-activated protein kinase (AMPK), which is responsible for the Alamandine-dependent NO increase in rat cardiomyocytes [11]. In the isolated and perfused goldfish heart, Alamandine exposure reduced the phosphorylation of Akt on Ser473, and of AMPKα on Thr172 (Figure 5A). This prompted us to verify the role of the cAMP-dependent protein kinase, PKA, recognized as a modulator of NOS activity [31]. Pre-treatment with the PKA antagonist KT5720 (10^−7^ M) abolished the stimulatory effect induced by Alamandine on the cardiac parameters (SV, SW, and CO) of the goldfish heart (Figure 5B).

### 3.5. Activation of the ACE2/Alamandine Axis under Hypoxia

**Figure 6:**
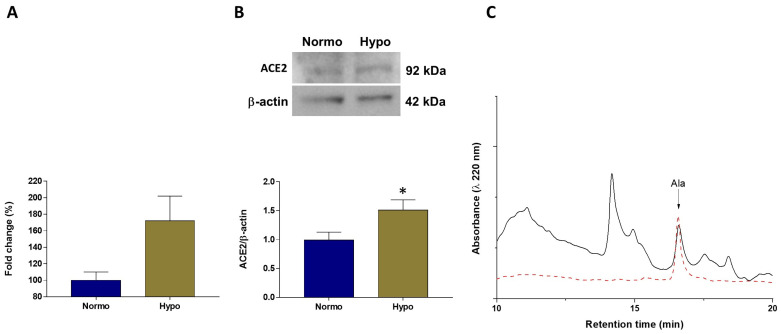
(**A**) Quantitative real-time PCR analysis of ace2 mRNA expression in cardiac extracts of goldfish *C. auratus* exposed to normoxia and hypoxia. Comparison of 2-ΔCt mean values of normoxic and hypoxic hearts, reported as percent fold change (*y*-axis). Statistic was assessed by one-way ANOVA followed by a Sidak’s multiple comparison test (*n* = 3); (**B**) Representative immunoblot and densitometric analysis of ACE2 expression in cardiac extracts of goldfish *C. auratus* exposed to normoxia and hypoxia. Data were expressed as means ± s.e.m. of absolute values from individual experiments (*n* = 3). Statistical analysis was performed by two-tailed unpaired *t*-test (* *p* < 0.05). (**C**) Representative HPLC chromatogram showing Alamandine (Ala) elution from plasma samples of goldfish *C. auratus* exposed to hypoxia compared with standard (red dotted line).

To evaluate whether exposure to hypoxia influences the ACE2/Alamandine axis, real-time PCR, Western Blotting, and HPLC analyses were performed on cardiac extracts and plasma of goldfish exposed to 4 days of hypoxia. Analysis of ace2 mRNA levels revealed an increasing trend in hypoxic vs. normoxic heart (~172% vs. 100%), although without statistical significance. However, comparative analyses of the blots showed a significant increase of ACE2 expression associated to hypoxia (Figure 6A,B). HPLC analysis also showed that, under hypoxia, plasma levels of Alamandine significantly increased by almost two-fold, reaching 59.6 ± 2.7 pmol/mL (mean ± s.e.m.) (Figure 6C).

**Figure 7:**
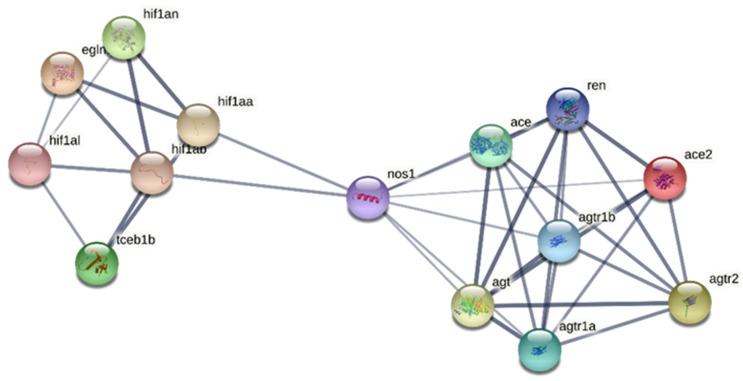
Analysis of the protein–protein interaction network (PPI) by the STRING online suite (version 11.5). The PPI network includes 14 proteins (number of nodes: 14; number of edges: 38; average node degree: 5.43; avg. local clustering coefficient: 0.623; expected number of edges: 2; PPI enrichment *p*-value: <1.0 × 10^−16^). ace2-related proteins: ace2, Angiotensin I converting enzyme 2; agtr2, Angiotensin II receptor, type 2; ace, Angiotensin I converting enzyme 1; agtr1a, Angiotensin II receptor, type 1a; agtr1b, Angiotensin II receptor, type 1b; ren, Renin; nos1, nitric oxide synthase; agt, Angiotensinogen; hif1-related proteins: egln1a, Egl-9 family hypoxia-inducible factor 1; hif1an, Hypoxia-inducible factor 1-alpha inhibitor; tceb1b, Transcription elongation factor B (SIII), polypeptide 1b; hif1al, Hypoxia-inducible factor 1, alpha subunit, -like; hif1ab, Hypoxia-inducible factor 1, alpha subunit b; hif1aa, Hypoxia-inducible factor 1, alpha subunit a.

In parallel, by STRING analysis in zebrafish (as the cyprinid model available in the STRING database) we observed that the network of interactions of ace2-related proteins includes nos1, and, through nos1, also hif1 alpha, plus other proteins related to hif1 and involved in the response to hypoxia (egln1a, Egl-9 family hypoxia-inducible factor 1; hif1an, Hypoxia-inducible factor 1-alpha inhibitor; tceb1b, Transcription elongation factor B (SIII), polypeptide 1b; hif1al, Hypoxia-inducible factor 1, alpha subunit, -like; hif1ab, Hypoxia-inducible factor 1, alpha subunit b) (Figure 7).

## 4. Discussion

In this study we provided the first in silico and in vitro evidence of the presence of a functional ACE2/Alamandine axis in the teleost fish *C. auratus*. We found that ACE2 is expressed in the goldfish heart and that a peptide with chemical properties similar to Alamandine is present in plasma. We also found that both ACE2 and Alamandine are sensitive to hypoxia. Under normoxia, the direct exposure of the isolated and perfused goldfish heart to exogenous Alamandine induced a dose-dependent increase of contractility which involves Mas-related receptors and the intracardiac NOS/NO system.

Homologous of ace2 [32], and ACE2 expression are documented in fish [33]. The fish protein shows a sequence identity with mammalian ACE2 from 55.1% to 60.5% (swamp eel and coelacanth, respectively) [33]. In the goldfish, ACE2 has a percentage of identity of about 57% with respect to the human and the mouse protein, and between 57.9% and 96.1% if compared to other fish species, such as thorny skate and carp, respectively [33]. Our sequence alignment by BLAST showed that the goldfish ace2 protein product (NCBI Acc. No. XP_026131313.1) is phylogenetically positioned in a cyprinid subcluster of proteins; this subcluster includes both ace2 and ace2-like isoforms. In agreement with the above in silico data, we showed that ACE2 gene product is expressed under normal conditions in several goldfish tissues, including the heart. In mammals, the enzyme is present in the cardiac tissue, although to a lesser extent compared to the intestine, which is considered a major ACE2-expressing region [34,35,36]. Very recently, in situ hybridization evidence in the zebrafish proposes that intestinal cells are the almost exclusive site for ACE2 expression [37]. Our data in goldfish, showing a strong expression of ace2 mRNA in the intestine, agree with this expression pattern. Moreover, the presence of both ace2 mRNA and protein in cardiac extracts suggests that the goldfish heart also possesses an intrinsic enzymatic ability towards ACE2 substrates (i.e., Ang A/Ang-(1-7)). Interestingly, by HPLC, we detected in goldfish plasma the presence of a peptide with elution properties similar to the Alamandine standard. This induced us to hypothesize that, under basal conditions, this cyprinid expresses an ACE2/Alamandine axis in which the heart represents a putative site for peptide generation, and the blood is the vehicle for its distribution to the periphery.

So far, information on the cardiovascular function of ACE2-generated peptides in fish is scarce. Available data are limited either to no effects, or to a mild hypotensive effect reported after Ang-(1–7) exposure in eel [38] and trout [39], respectively, but no information is currently available on the cardiovascular effects of Alamandine in fish. Here we showed that Alamandine, exogenously administered, modulates the isolated and spontaneously beating goldfish heart, perfused under normal conditions. It elicited cardiostimulation, revealed by the dose-dependent enhancement of SV, SW, and CO, while it did not affect HR. This positive effect was significant from 10^−11^ M and persisted up to the highest concentrations tested. In mammals, the peptide ameliorates hemodynamic performance after ischemia/reperfusion [10], suppresses AngII-dependent hypertrophy [11], and prevents myocyte hypertrophy and cardiac fibrosis induced by aortic constriction [40]. It also enhances contractility in cardiomyocytes from hypertensive rats [29]. Our data suggest that, under basal conditions, Alamandine is generated and controls the cardiac performance, adding its activity to that elicited by AngII [15,16,17,18]. This stimulates research to clarify the functional significance of a multiple RAS-mediated control of the fish heart. It also opens the way to evaluate whether and to what extent, according to the mammalian model [41,42], the cardiac modulation induced by Alamandine also represents a “protective limb” of the RAS in fish.

In mammals, the Alamandine-dependent cardiovascular effects require binding with MrgD receptors [9,42]. These receptors belong to the family of the Mas receptors [43], which are able to bind another peptide of the alternative RAS pathway, Ang-(1-7) [7]. We observed that the cardio-stimulatory effect elicited by Alamandine is abolished by the pretreatment with the MrgD and Mas receptor antagonist D-Pro7-Ang-(1-7) (10^−12^ M), and with the Mas selective antagonist, D-Ala7-Ang-(1-7) (10^−11^ M), suggesting that both MrgD and Mas receptors are part of the functional axis activated by Alamandine in the goldfish heart. However, they contrast with the current knowledge that suggests MrgD as the exclusive receptor for Alamandine, at least in mammals [44], and indicates Mas as late receptors, appeared in the evolutive lineage only after bony fish [45]. Further studies are needed to clarify this issue by taking into account the complexity of protein evolution and the event of genome duplication occurring in teleost fish, whose consequence is the expression of an elevated number of proteins that possibly perform similar functions [46]. If the absence of Mas receptors is confirmed in teleost, our physio-pharmacological evidence that Alamandine-induced cardiostimulation is counteracted by both MrgD and Mas antagonists may indicate in the goldfish the presence of an “ancestral” protein with functional traits common to both receptor types. Data in mammals show that both MrgD and Mas receptors activate downstream pathways mediated by PKA-dependent signaling and that involve the NOS/NO system [47,48]. In rodents, Alamandine, via an MrgD-dependent NO release, stimulates cardiomyocyte contractility and suppresses AngII-induced cardiomyocyte hypertrophy [11,29]. We showed here that the positive effects of Alamandine are counteracted by inhibiting NOS activity via L-NMMA and that, after exposure to the peptide, the cardiac expression of an active phosphorylated eNOS-like isoform increased. This suggested that the NOS activation is involved in the cascade elicited by Alamandine to stimulate the heart. In the goldfish, micromolar concentrations of a NO donor exert a mild basal negative inotropism, while endogenous NOS-derived NO improves the performance of the challenged heart, as in the case of preload enhancement (i.e., the Frank–Starling response; [25]), and of low oxygen availability [25,49,50]. The putative involvement of NO in the cardio-stimulatory effects induced by Alamandine in the goldfish heart, further fuels the debate on the different cardiac biological functions of the NOS/NO system in fish. Available evidence suggests that different and/or opposite effects can be elicited by NO depending on the types of stimulation, the spatial confinement of NOSs within myocardiocytes, the substrate availability, the amount of NO generated, as well as the recruitment of distinct downstream pathways (for references, see [51]).

In mammals eNOS undergoes regulation via multi-site phosphorylation involving numerous kinases, including AMPK, Akt, and PKA (for extensive review see [52]). Here we found that cardiac AMPK and AKT phosphorylation decreases in the presence of Alamandine. Accordingly, it is improbable that the activation of these kinases positively modulates the goldfish cardiac NOS enzyme. Data in mammals show that the role of these kinases in the cardiac effects induced by Alamandine is under debate, with the peptide showing no effect on Akt phosphorylation on Ser473, while stimulating AMPK phosphorylation (at Thr172) [11]. Interestingly, we observed that the hemodynamic stimulation elicited by Alamandine on the perfused goldfish heart is suppressed when PKA is inhibited by KT5720. The possibility exists that, in the presence of Alamandine, PKA may induce NOS activation with consequent intracardiac NO generation. This is supported by the ability of this kinase to directly activate eNOS via phosphorylation of Ser635 and Ser1179 residues [31].

Of note, when the effects of Alamandine were analyzed in the presence of specific inhibitors, a non-significant tendency to depress the cardiac parameters became evident. Whether, in the goldfish, Alamandine induces multiple and even divergent effects, probably masked by the dominant cardiostimulation, cannot be excluded. This may stimulate further ad hoc investigations designed by considering the complexity of this novel and poorly explored RAS limb, the dynamic balance between the cardiac effects induced by the different RAS components, as well as the complex network of downstream intracellular cascades.

Cyprinids, including the goldfish *C. auratus*, are known for their extraordinary physiological capacity to improve heart performance under O_2_ deprivation [25,53]. In the goldfish, this response involves many mediators, including NO, and Hif1α [25,54]. We analyzed whether in the goldfish the ACE2/Alamandine axis is sensitive to low O_2_. The qRT-PCR, Western Blotting, and HPLC data showed that both cardiac ACE2 and plasma Alamandine levels are upregulated when goldfish are exposed to prolonged environmental hypoxia. In addition, by using as a reference the ace2 protein of a putative model of another Cyprinid/teleost species (the zebrafish), together with seven proteins functionally related to ace2 itself, we found by STRING analysis that the network of proteins interacting with ace2 is intertwined with crucial determinants of the hypoxia-related responsiveness, such as hif1a, and occurs via nos1. The NOS-HIF interaction is well described in mammals [55,56,57] and in teleost fish [25,58,59]. In mammals, during hypoxic stress, as that occurring under ischemia [60,61,62], HIF-1α via activation of several critical genes [63], including NOS [64], significantly contributes to cell survival. In the goldfish heart, HIF-1α and eNOS protein levels are significantly increased under hypoxia [25], suggesting that HIF-1α and its probable cross-talk with NOS may participate in the hypoxia-elicited cardio-protective responses [25]. So far, the analysis of the NOS system in teleost has provided a complex picture. Together with the Western Blotting data presented here, physio-pharmacological and immunolocalization studies detected an eNOS-like activity in the heart of several teleost [17,51,60,61,62]. However, a gene for canonical eNOS (NOS3) is not reported in fish, contrary to the nNOS (NOS1) and iNOS (NOS2) genes [63]. It was proposed that in fish a set of NOS proteins may cover the functional traits of eNOS [51,63]. In particular, Cyprinids show a trend to increase the number of Nos-type proteins, with NOS1-type proteins more closely related to NOS3 than to NOS2 [64]. In this respect, the possibility exists that in the goldfish heart NOS1 may have evolved to support eNOS-like functions.

The in silico evidence on the network of ace2-interacting proteins reported in the present study deserve assessment in an in vitro setting, to evaluate whether teleost cardiac NOS enzymes, and a possible cross-talk with HIF, may represent functional nodes in the ace-related response to hypoxia.

## 5. Conclusions

The results of the present study proposed that an ACE2/Alamandine axis is present in the goldfish *C. auratus* and is able to elicit stimulatory effects on the basal heart performance, by involving a receptor still to be characterized, with functional traits resembling those of both MrgD and Mas receptors. Our data, although preliminary, also suggested that these effects involve the NOS/NO system. This expands the role of NO as an intracardiac orchestrator of the humoral modulation of the fish heart. Further analyses are needed for a complete cause–effect relationship between Alamandine and the nitrergic system, and for a complete characterization of the specific receptor for Alamandine expressed in the goldfish. The exploration of the physiological significance of the ACE2/Alamandine axis in fish is still at its beginning. Many questions remain open, including the conditions leading to the activation of the ACE2/Alamandine axis, the true nature of the membrane receptor recruited by Alamandine, and the subsequent NO-dependent and/or independent intracellular signaling. In addition, the biological significance of this cardiac modulation in the larger context of the RAS-mediated control of the teleost heart requires to be elucidated. Further information on the above aspects is welcome, not only for improving the knowledge on the molecular machinery that controls cardiac homeostasis in fish, but also to better understand the mechanisms that provide adaptive flexibility to the heart in response to environmental stress. Our preliminary data linking the ACE2/Alamandine axis, and the nitrergic system with hypoxia encourage research in this direction.

## Figures and Tables

**Table 1:** Features of primer sequences for real-team PCR expression analysis.

*Gene*	*RefSeq mRNA*	*Sense Primer 5′-3′* *(Tm)*	*Antisense Primer 5′-3′* *(Tm)*	*PCR Size* *(bp)*
**ace2**	XM_026275528.1	GAAATGAATTTCAAGCCAGAG (58 °C)	GACTGCGTCTGCTTTGGT (55 °C)	121
**28S RNA**	EF417169.1	GGTCTAAGTCCTTCTGAT (51 °C)	GGCTGCATTCCCAAACAAC (54 °C)	112

**Table 2:** Baseline hemodynamic parameters of the isolated and perfused goldfish heart (*n* = 6).

Heart Rate (bpm)	Cardiac Output (mL/min/Kg)	Stroke Volume (mL/Kg)	Stroke Work (mJ/g)
75.50 ± 3.334	13.55 ± 0.673	0.18 ± 0.013	0.24 ± 0.022

## Data Availability

All data used to support the findings of this study are included within the article and Supplementary Materials.

## Supplementary Materials

The following supporting information can be downloaded at: https://www.mdpi.com/article/10.3390/antiox11040764/s1, Figure S1. Chromatograms showing Alamandine standard at different concentrations. Figure S2. Amino acid sequence alignment of teleost ace2 and ace2-like protein isoforms by NCBI BLAST. The phylogenetic tree (built with Neighbor Joining method) comprises 95 sequences from the result of processing (by BLASTP) the ace2 reference protein sequence of C. auratus (Acc. N. XP_026131313.1) for matching the refseq_protein database, subset of Teleostei (taxid: 32443).
