# Supplementary material for: An ACE2-Alamandine Axis Modulates the Cardiac Performance of the Goldfish Carassius auratus via the NOS/NO System
Source: Antioxidants (Basel). 2022 Apr 12;11(4):764. doi: 10.3390/antiox11040764 (PMC9026556; doi:10.3390/antiox11040764)
Supplement: Supplementary file 1 [file antioxidants-11-00764-s001.zip › antioxidants-1652286-supplementary.pdf]

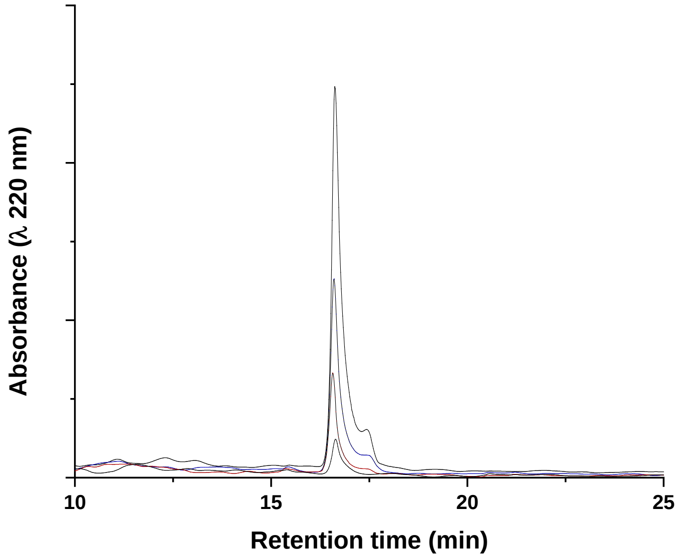

Supplementary Figure S1. Chromatograms showing Alamandine standard at different concentrations.

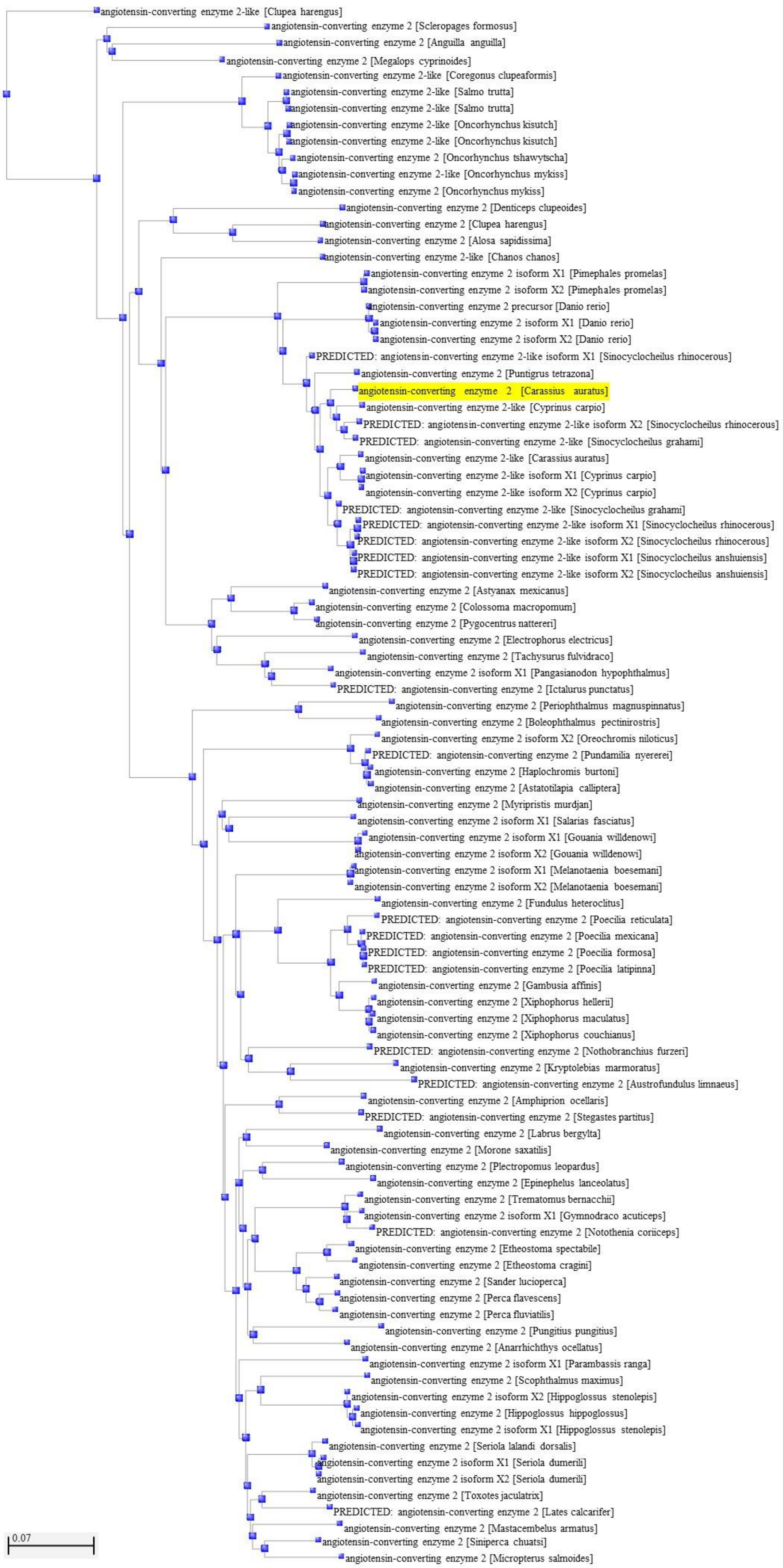
angiotensin-converting enzyme 2-like [Clupea harengus]
angiotensin-converting enzyme 2 [Scleropages formosus]
angiotensin-converting enzyme 2 [Anguilla anguilla]
angiotensin-converting enzyme 2 [Megalops cyprinoides]
angiotensin-converting enzyme 2-like [Coregonus clupeaformis]
angiotensin-converting enzyme 2-like [Salmo trutta]
angiotensin-converting enzyme 2-like [Salmo trutta]
angiotensin-converting enzyme 2-like [Oncorhynchus kisutch]
angiotensin-converting enzyme 2-like [Oncorhynchus kisutch]
angiotensin-converting enzyme 2 [Oncorhynchus tshawytscha]
angiotensin-converting enzyme 2-like [Oncorhynchus mykiss]
angiotensin-converting enzyme 2 [Oncorhynchus mykiss]
angiotensin-converting enzyme 2 [Denticeps clupeoides]
angiotensin-converting enzyme 2 [Clupea harengus]
angiotensin-converting enzyme 2 [Alosa sapidissima]
angiotensin-converting enzyme 2-like [Chanos chanos]
angiotensin-converting enzyme 2 isoform X1 [Pimephales promelas]
angiotensin-converting enzyme 2 isoform X2 [Pimephales promelas]
angiotensin-converting enzyme 2 precursor [Danio rerio]
angiotensin-converting enzyme 2 isoform X1 [Danio rerio]
angiotensin-converting enzyme 2 isoform X2 [Danio rerio]
PREDICTED: angiotensin-converting enzyme 2-like isoform X1 [Sinocyclocheilus rhinocerous]
angiotensin-converting enzyme 2 [Puntigrus tetrazona]
angiotensin-converting enzyme 2 [Carassius auratus]
angiotensin-converting enzyme 2-like [Cyprinus carpio]
PREDICTED: angiotensin-converting enzyme 2-like isoform X2 [Sinocyclocheilus rhinocerous]
PREDICTED: angiotensin-converting enzyme 2-like [Sinocyclocheilus grahami]
angiotensin-converting enzyme 2-like [Carassius auratus]
angiotensin-converting enzyme 2-like isoform X1 [Cyprinus carpio]
angiotensin-converting enzyme 2-like isoform X2 [Cyprinus carpio]
PREDICTED: angiotensin-converting enzyme 2-like [Sinocyclocheilus grahami]
PREDICTED: angiotensin-converting enzyme 2-like isoform X1 [Sinocyclocheilus rhinocerous]
PREDICTED: angiotensin-converting enzyme 2-like isoform X2 [Sinocyclocheilus rhinocerous]
PREDICTED: angiotensin-converting enzyme 2-like isoform X1 [Sinocyclocheilus anshuiensis]
PREDICTED: angiotensin-converting enzyme 2-like isoform X2 [Sinocyclocheilus anshuiensis]
angiotensin-converting enzyme 2 [Astyanax mexicanus]
angiotensin-converting enzyme 2 [Colossoma macropomum]
angiotensin-converting enzyme 2 [Pygocentrus nattereri]
angiotensin-converting enzyme 2 [Electrophorus electricus]
angiotensin-converting enzyme 2 [Tachysurus fulvidraco]
angiotensin-converting enzyme 2 isoform X1 [Pangasianodon hypophthalmus]
PREDICTED: angiotensin-converting enzyme 2 [Ictalurus punctatus]
angiotensin-converting enzyme 2 [Periophthalmus magnuspinnatus]
angiotensin-converting enzyme 2 [Boleophthalmus pectinirostris]
angiotensin-converting enzyme 2 isoform X2 [Oreochromis niloticus]
PREDICTED: angiotensin-converting enzyme 2 [Pundamilia nyererei]
angiotensin-converting enzyme 2 [Haplochromis burtoni]
angiotensin-converting enzyme 2 [Astatotilapia calliptera]
angiotensin-converting enzyme 2 [Myripristis murdjan]
angiotensin-converting enzyme 2 isoform X1 [Salarias fasciatus]
angiotensin-converting enzyme 2 isoform X1 [Gouania willdenowi]
angiotensin-converting enzyme 2 isoform X2 [Gouania willdenowi]
angiotensin-converting enzyme 2 isoform X1 [Melanotaenia boesemani]
angiotensin-converting enzyme 2 isoform X2 [Melanotaenia boesemani]
angiotensin-converting enzyme 2 [Fundulus heteroclitus]
PREDICTED: angiotensin-converting enzyme 2 [Poecilia reticulata]
PREDICTED: angiotensin-converting enzyme 2 [Poecilia mexicana]
PREDICTED: angiotensin-converting enzyme 2 [Poecilia formosa]
PREDICTED: angiotensin-converting enzyme 2 [Poecilia latipinna]
angiotensin-converting enzyme 2 [Gambusia affinis]
angiotensin-converting enzyme 2 [Xiphophorus hellerii]
angiotensin-converting enzyme 2 [Xiphophorus maculatus]
angiotensin-converting enzyme 2 [Xiphophorus couchianus]
PREDICTED: angiotensin-converting enzyme 2 [Nothobranchius furzeri]
angiotensin-converting enzyme 2 [Kryptolebias marmoratus]
PREDICTED: angiotensin-converting enzyme 2 [Austrofundulus limnaeus]
angiotensin-converting enzyme 2 [Amphiprion ocellaris]
PREDICTED: angiotensin-converting enzyme 2 [Stegastes partitus]
angiotensin-converting enzyme 2 [Labrus bergylta]
angiotensin-converting enzyme 2 [Morone saxatilis]
angiotensin-converting enzyme 2 [Plectropomus leopardus]
angiotensin-converting enzyme 2 [Epinephelus lanceolatus]
angiotensin-converting enzyme 2 [Trematomus bernacchii]
angiotensin-converting enzyme 2 isoform X1 [Gymnodraco acuticeps]
PREDICTED: angiotensin-converting enzyme 2 [Notothenia coriiceps]
angiotensin-converting enzyme 2 [Etheostoma spectabile]
angiotensin-converting enzyme 2 [Etheostoma cragini]
angiotensin-converting enzyme 2 [Sander lucioperca]
angiotensin-converting enzyme 2 [Perca flavescens]
angiotensin-converting enzyme 2 [Perca fluviatilis]
angiotensin-converting enzyme 2 [Pungitius pungitius]
angiotensin-converting enzyme 2 [Anarrhichthys ocellatus]
angiotensin-converting enzyme 2 isoform X1 [Parambassis ranga]
angiotensin-converting enzyme 2 [Scophthalmus maximus]
angiotensin-converting enzyme 2 isoform X2 [Hippoglossus stenolepis]
angiotensin-converting enzyme 2 [Hippoglossus hippoglossus]
angiotensin-converting enzyme 2 isoform X1 [Hippoglossus stenolepis]
angiotensin-converting enzyme 2 [Seriola lalandi dorsalis]
angiotensin-converting enzyme 2 isoform X1 [Seriola dumerili]
angiotensin-converting enzyme 2 isoform X2 [Seriola dumerili]
angiotensin-converting enzyme 2 [Toxotes jaculatrix]
PREDICTED: angiotensin-converting enzyme 2 [Lates calcarifer]
angiotensin-converting enzyme 2 [Mastacembelus armatus]
angiotensin-converting enzyme 2 [Siniperca chuatsi]
angiotensin-converting enzyme 2 [Micropterus salmoides]
0.07

Supplementary Figure S2. Amino acid sequence alignment of teleost ace2 and ace2-like protein isoforms by NCBI BLAST. The phylogenetic tree (built with Neighbor Joining method) comprises 95 sequences from the result of processing (by BLASTP) the ace2 reference protein sequence of *C. auratus* (Acc. N. XP_026131313.1) for matching the *refseq_protein* database, subset of Teleostei (taxid: 32443).
